# The Effect of *Vitex Agnus - Castus* Plant on Some Markers of Oxidative Stress, Lipid Profile and Insulin Resistance in Women with Polycystic Ovary Syndrome: A Randomized, Double-Blind Controlled Clinical Trial Study

**DOI:** 10.5935/1518-0557.20250165

**Published:** 2026

**Authors:** Aniseh Hatami, Fateme Seidi, Ali Khosrowbeygi, Azam Moslemi, Farideh Jalali-Mashayekhi

**Affiliations:** 1 Student Research Committee, Arak University of Medical Sciences, Arak, Iran; 2 Department of Obstetrics and Gynecology, School of Medicine, Arak University of Medical Sciences, Arak, Iran; 3 Department of Biochemistry and Genetics, School of Medicine, Arak University of Medical Sciences, Arak, Iran; 4 Molecular and Medicine Research Center, Arak University of Medical Sciences, Arak, Iran; 5 Department of Biostatistics, School of Medicine, Arak University of Medical Sciences, Arak, Iran

**Keywords:** polycystic ovary syndrome, Vitex agnus-castus, oxidative stress, lipid profile, insulin resistance, randomized controlled trial

## Abstract

**Objective:**

This randomized controlled trial investigated the efficacy of standardized *Vitex agnus-castus* extract in managing features of PCOS. The primary aim was to assess changes in oxidative stress markers; secondary outcomes included lipid profile, insulin resistance, and clinical signs such as hirsutism and menstrual frequency.

**Methods:**

Sixty women with PCOS were randomly assigned to a *Vitex* group (5.8 mg daily, standardized to 0.42-0.82 mg Aucubin) or placebo for 12 weeks. Dietary habits and physical activity and physical activity were maintained throughout the study. Serum total antioxidant capacity, glutathione peroxidase, reduced glutathione, and other biochemical and clinical parameters were assessed preand post-intervention. Between-group differences were analyzed using independent t-tests and ANCOVA.

**Results:**

Compared to placebo, Vitex significantly increased total antioxidant capacity (effect size = 13.01), glutathione peroxidase (3.35), reduced glutathione (3.88), total thiol (3.34), and HDL (5.74) (all *p*<0.05). It decreased total oxidant status (-6.49), oxidative stress index (-9.30), malondialdehyde (-5.29), fasting blood sugar (-5.10), HOMA-IR (-0.31), LDL (-2.85), ALT (-3.51), and mFG score (-5.38). Menstrual frequency improved (3.51), and left ovarian volume reduced (-0.80).

**Conclusions:**

*Vitex agnus-castus* improved oxidative stress markers and insulin resistance and favorably modulated clinical manifestations of PCOS. These findings suggest a clinically meaningful benefit and support further investigation into Vitex as an adjunctive therapy.

## INTRODUCTION

Polycystic ovary syndrome (PCOS) is a prevalent endocrine disorder affecting 6-22% of women of reproductive age (Bargiota & Diamanti-Kandarakis, 2012). Defined by the Rotterdam criteria and endorsed by the 2018 International Evidence-based Guideline, PCOS diagnosis requires the presence of at least two of the following: hyperandrogenism (biochemical and/or clinical), chronic anovulation and menstrual dysfunction, and polycystic ovarian morphology on ultrasonography, after excluding other endocrine disorders (Bargiota & Diamanti-Kandarakis, 2012; Siddiqui *et al.*, 2022). This complex disorder poses a significant health burden, increasing the risk of metabolic and cardiovascular complications, including insulin resistance, obesity, dyslipidemia, gestational diabetes, type 2 diabetes, and cardiovascular disease (Vink *et al.*, 2006).

Although the exact etiology of PCOS remains incompletely understood, it is widely recognized that a complex interplay of genetic, hormonal, and environmental factors contributes to its pathogenesis (Vink *et al.*, 2006; Bednarska & Siejka, 2017; Siddiqui *et al.*, 2022). Among these, oxidative stress has emerged as a critical player in the pathophysiology of PCOS (Bednarska & Siejka, 2017). Insulin resistance and hyperglycemia, which are frequently observed in women with PCOS, are key drivers of elevated oxidative stress (Bannigida *et al.*, 2020). Conversely, oxidative stress exacerbates insulin resistance, creating a vicious cycle that fuels hyperandrogenism, hyperinsulinemia, dyslipidemia, and an altered luteinizing hormone/follicle-stimulating hormone ratio, ultimately contributing to PCOS development and progression (Zeng *et al.*, 2020). Recent PCOS interventions include lifestyle changes (diet, exercise) and more targeted drug therapies (metformin, Glucagon-like peptide-1 agonists, inositols). Individualized, multifaceted approaches integrating lifestyle and medication are increasingly emphasized for successful management of this chronic condition. The complex and multifactorial nature of PCOS poses significant challenges for its clinical management. Beyond herbal interventions, several recent studies have expanded our understanding of PCOS treatment by emphasizing the role of oxidative stress and inflammation in clinical outcomes, especially in the context of assisted reproductive technologies (ART). A systematic review by Moreira *et al*. compared the follicular fluid composition between women with PCOS and normo-ovulatory women, revealing significant differences in oxidative stress and inflammatory biomarkers that may adversely affect ART outcomes (Moreira *et al.*, 2023). Furthermore, a study by Vale-Fernandes *et al*. identified a positive association between elevated anti-Müllerian hormone (AMH) levels and increased oxidative stress in the follicular fluid of women with PCOS, suggesting that AMH may serve as a surrogate marker for oxidative imbalance and potential reproductive compromise. These findings underscore the importance of targeting oxidative stress in PCOS not only for metabolic and endocrine improvement but also for optimizing fertility-related outcomes (Vale-Fernandes *et al.*, 2024).

Given the well-established role of oxidative stress in PCOS pathogenesis and its associated complications, therapeutic strategies aimed at restoring oxidative balance hold considerable promise. In this context, herbal medicines have garnered increasing attention due to their long history of traditional use and perceived lower incidence of adverse effects compared to conventional pharmaceuticals (Li *et al.*, 2022; Masjedi *et al.*, 2024). Several studies have reported the benefits of herbal interventions in women’s health, including improvements in oxidative stress status related to premenstrual syndrome and infertility (Li *et al.*, 2022; Puglia *et al.*, 2023).

One such herbal remedy is *Vitex agnus-castus* (*Vitex*), a medicinal plant widely used for managing various gynecological conditions, such as menopausal symptoms, menstrual disorders, and mastalgia (Shayan *et al.*, 2016; Wang *et al.*, 2018). Phytochemical analyses of *Vitex* extracts have identified a range of bioactive compounds with antioxidant properties, including vanillic acid, luteolin, quercetin, caffeic acid, resveratrol, and naringenin (Kavaz *et al.*, 2022). Preclinical and clinical studies suggest that *Vitex* may modulate menstrual irregularities and hyperandrogenism, both of which are hallmark features of PCOS (Alois & Estores, 2019). Despite these promising findings, robust clinical trials evaluating the efficacy of *Vitex* specifically for PCOS management remain limited.

To address this gap, we conducted a randomized, placebo-controlled trial to investigate the effects of a standardized *Vitex* extract on key parameters relevant to PCOS. Specifically, we evaluated its impact on oxidative stress markers (total antioxidant capacity, glutathione peroxidase, reduced glutathione, total thiol, total oxidant capacity, oxidative stress index, and malondialdehyde), lipid profile (cholesterol, low-density lipoprotein, and high-density lipoprotein), insulin resistance, ovarian volume, hirsutism score, and menstrual frequency in women with PCOS. This study aims to provide rigorous evidence regarding the therapeutic potential of *Vitex* in the management of this complex and multifactorial condition.

## MATERIAL AND METHODS

### Study registration and approval

This study was a double-blind, placebo-controlled, parallel-group clinical trial. The trial was conducted in accordance with the Declaration of Helsinki and was carried out from April 2023 to January 2024 in Ayatollah Taleghani Educational and Therapeutic Center, Arak, Iran. This study was reviewed and deemed exempt from ethics approval by the Ethics Committee of Arak University of Medical Sciences with the reference number: IR.ARAKMU.REC.1401.333, dated February 12th, 2023. The study was also registered in the Iranian Registry of Clinical Trials (registration code: IRCT20230222057493N1) on March 28, 2023. This study was conducted using CONSORT reporting guidelines. Both participants and outcome assessors were blinded to group assignments throughout the study. Written informed consent was obtained from all participants prior to their enrollment, and the consent form was approved by the Ethics Committees of Arak University of Medical Sciences, Iran.

### Population (Inclusion and exclusion criteria)

Women aged 18-45 years diagnosed with PCOS according to the Rotterdam criteria were recruited. Inclusion criteria were: (1) PCOS diagnosis per Rotterdam criteria; (2) age 18-45 years; and (3) no intention to become pregnant during the study. Exclusion criteria were: (1) pregnancy or breastfeeding; (2) smoking; (3) hyperprolactinemia; (4) thyroid disorders; (5) congenital adrenal hyperplasia; (6) diabetes mellitus; (7) use of antioxidant or herbal supplements within the past three months; and (8) use of dopamine antagonists.

### Sample size

Using GSH as a variable (Shokrpour & Asemi, 2019), and a two-sided significance level (α) of 0.05 and a power (1-β) of 80% (β = 0.20), according to the following equation, the minimum sample size was calculated 28 for each group:


$$\begin{array}{rl} & n\geq 2{\left(Z_{a}+Z_{B}\right)}^{2}\sigma^{2}/{\left({µ}_{1}-{µ}_{2}\right)}^{2},\\ & a=0.05\;\beta =0.20\;{µ}_{1}=483.8\;{µ}_{2}=519.4\;\sigma \approx \end{array}$$

Accounting for an estimated 10% dropout rate, we aimed to recruit 30 participants per group.

### Randomization

#### Sequence generation

A randomization list was created prior to the commencement of the study. The permuted block randomization method with blocks of four was employed in this study. The *Vitex* group was labelled as A, and the placebo group as B. Six possible combinations-AABB, BBAA, BABA, ABBA, BAAB, and ABAB-were written on separate sheets of paper and placed into a container. Each time, one sheet was randomly drawn from the container, the combination on it was recorded, and the sheet was returned to the container. Given the sample size of 60, this process was repeated 15 times, with each combination being recorded sequentially. Subsequently, each letter (A or B) in the recorded sequence was assigned a number from one to 60.

#### Allocation concealment mechanism and blinding

Letters were individually placed into envelopes, each marked with a specific number. When a participant was recruited, an envelope was opened, and the number indicated on it was used to allocate the subject to either the *Vitex* group or the placebo group. Subjects and healthcare providers were blinded and unaware of the study grouping. A.M. generated the random allocation sequence, F.S. enrolled participants, and A.H. assigned participants to *Vitex* and placebo groups.

### Intervention

Following randomization, participants were assigned to either the *Vitex* group (n=30) or the placebo group (n=30) for a 12-week intervention period. Participants were instructed to maintain their usual dietary habits without any modifications throughout the study period. They were explicitly advised against initiating any new dietary regimens, herbal medications, antioxidant supplements, during the 12-week intervention. Adherence to these guidelines was continuously monitored through telephone follow-ups. At the baseline visit and the end of the 12-week intervention, after a 12-hour overnight fast, 10 mL of venous blood was collected. Participants in the *Vitex* group received 5.8 mg daily of a standardized *Vitex* extract (standardized to 0.42-0.82 mg Aucubin) administered orally as *Agnugol* tablets (Goldaro Pharmaceutical Company, Isfahan, Iran) and this standardization was carried out according to the production and quality control processes of the mentioned company. The placebo group received matching placebo tablets containing cellulose acetate (Goldaro Pharmaceutical Company, Isfahan, Iran). All tablets were identical in appearance (shape, color, and smell) and were packaged in identical containers. During this study, patients were not deprived of the main medicine, which included metformin and an oral contraceptive (3 mg Drospirenone + 0.03 mg Ethinyl estradiol).

### Laboratory Methods

#### Sample Collection and Preparation

Venous blood samples were collected from all participants after a 12-hour overnight fast. Samples were centrifuged at 3000 rpm for 10 minutes, and the resulting serum was stored at -80°C until analysis.

#### Biochemical Assays

Serum total thiol (TT) levels were measured using Ellman’s reagent (DTNB) (Eyer *et al.*, 2003). Total antioxidant capacity (TAC) was quantified using the ferric reducing ability of plasma (FRAP) assay (Samimi *et al.*, 2024). Total oxidant status (TOS) was assessed by the ferric-xylenol orange method (Erel, 2005).

Catalase (CAT) activity was determined by incubating serum with hydrogen peroxide as a substrate, with the enzymatic reaction terminated by ammonium molybdate (Samimi *et al.*, 2024). Commercially available kits were used to evaluate activities of GPx and levels of GSH (Novin Navand Salamat Pishtaz Co. Urmia, Iran), and MDA (Malondialdehyde) (Kushan Zist Azma Parseh Co. Tehran, Iran).

Fasting blood sugar (FBS), triglycerides (TG), total cholesterol (TC), high-density lipoprotein cholesterol (HDL-C), low-density lipoprotein cholesterol (LDL-C), aspartate aminotransferase (AST), and alanine aminotransferase (ALT) levels were measured using an automated analyzer (Hitachi 717, Japan) and commercially available enzymatic kits (Delta Darman Part, Iran). Serum insulin levels were determined by enzyme-linked immunosorbent assay (ELISA) using a commercial kit (Monobind, Iran). Insulin resistance was calculated using the homeostasis model assessment of insulin resistance (HOMA-IR) index, as follows (Tahapary *et al.*, 2022):

Fasting insulin (µU/dL) × Fasting blood glucose (mmol/L) / 22.5.

A HOMA-IR value ≥2.5 was considered indicative of insulin resistance (Minh *et al.*, 2021).

#### Outcome measures

The primary outcome of this study was the change in oxidative stress markers, specifically TAC, GPx, GSH, TOS, oxidative stress index (OSI), and MDA levels. Secondary outcomes included changes in lipid profile (TG, TC, HDL-C, LDL-C), insulin resistance (HOMA-IR), ovarian volume assessed by ultrasonography, menstrual frequency, and hirsutism score measured using the Modified Ferriman-Gallwey (mFG) scoring system.

The mFG score (Willis *et al.*, 2020) was used to quantify the degree of hirsutism, and ultrasonography was used to measure the ovary volume. Menstrual frequency was calculated by dividing the observed number of menstrual periods during the 12-week intervention by the expected number of periods (n=3, assuming a regular 28-day cycle). The menstrual frequency was then compared between the *Vitex* and placebo groups (Kiel *et al.*, 2020).

### Statistical analysis

Statistical analysis was performed using SPSS version 23.0 (IBM, New York, USA). The results of demographic factors were reported as Mean±SD, and the results of other variables were reported as Mean±standard error of the mean (SEM). The effect sizes (Cohen’s d) were calculated to assess the magnitude of differences, beyond statistical significance. A *p*-value of less than 0.05 was considered statistically significant.

Normality of continuous variables was assessed using the Shapiro-Wilk test. Within-group changes from baseline to the end of the intervention were analyzed using paired t-tests. Independent samples t-tests were used to compare changes between the *Vitex* and placebo groups. Analysis of covariance (ANCOVA) was employed to adjust for baseline differences between groups when comparing outcomes at the end of the intervention.

## RESULTS

**Figure 1:**
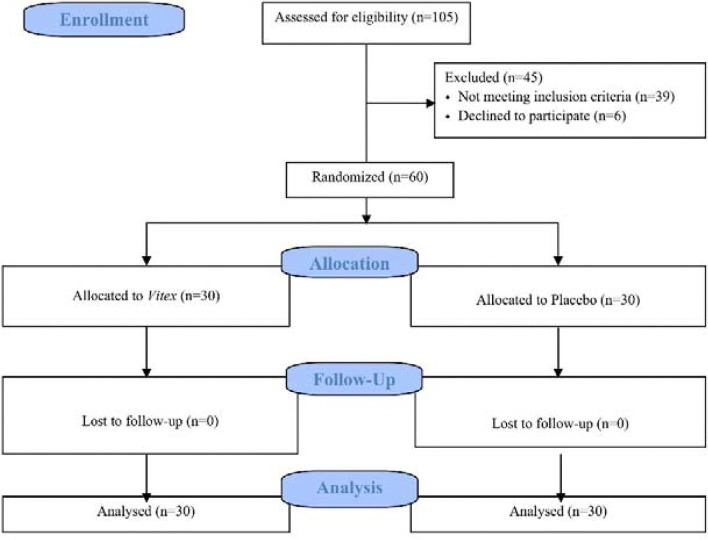
Consort diagram of the study population.

One hundred and one women with PCOS were selected using convenience sampling. Thirty-nine subjects were excluded due to exclusion criteria, six subjects were excluded because they were not satisfied to participate in the study, and finally, 60 subjects were included in the study. These subjects were divided into two groups: *Vitex* and placebo, using the permuted block randomization method with blocks of four. None of the 60 subjects who were included in the study were subsequently excluded, and all remained in the study until its completion (Figure 1). The recruitment period began on April 4, 2023 and ended on January 20, 2024.

Table 1 presents the baseline and post-intervention anthropometric characteristics of the participants in both the *Vitex* and Placebo groups. At baseline, there were no statistically significant differences between the two groups in terms of age (*p*=0.87), height (*p*=0.88), weight (*p*=0.68), or BMI (*p*=0.76). This indicates that the groups were well-matched at the start of the study. After the intervention period, there were no statistically significant differences between the *Vitex* and Placebo groups in terms of weight (*p*=0.57) or BMI (*p*=0.82).

**Table 1:** Baseline and post-intervention anthropometric characteristics.

Variables	Vitex (n=30)	Placebo (n=30)	*p*-value^†^
Age (year)	26.37±5.53	26.60±5.71	0.87
Height (m)	1.60±0.06	1.63±0.06	0.88
Weight baseline (Kg)	66.86±11.80	68.20±13.26	0.68
Weight post-intervention (Kg)	65.70±10.41	67.30±11.60	0.57
BMI baseline (Kg/m^2^)	26.09±4.87	25.70±5.26	0.76
BMI post-intervention (Kg/m^2^)	25.64±4.43	25.37±4.68	0.82

Notes: Results are presented as Mean±SD.

† Independent t-test.

Adjusted *p*-values corrected using Bonferroni correction.

Tables 2 and 3 demonstrate that comparisons were made between and within groups at baseline and the end of the trial. Findings from the independent t-test at the trial’s conclusion showed that the consumption of *Vitex* significantly increased the levels of TAC (*p*<0.001), GPx (*p*<0.05), GSH (*p*<0.05), TT (*p*<0.05), HDL (*p*<0.001) and Menstrual frequency (0.046).

**Table 2:** Comparison of the effects of Vitex and placebo consumption on biochemical characteristics of women with PCOS.

Variables	Vitex (n=30)	Placebo (n=30)	p-value^†^	Effect size (Cohen’s d)	*p*-value ^§^
**TAC (mM)** Before After	0.60±0.02 1.10±0.04	0.65±0.02 0.64±0.03	0.09 0.001	-2.5 13.01	0.001
*p*-value^‡^	0.001	0.55			
**TOS (µM)** Before After	11.76±0.55 9.13±0.52	13.65±0.94 14.18±0.97	0.09 0.001	-2.45 -6.48	0.001
*p*-value^‡^	0.001	0.58			
OSI % Before After	2.00±0.10 0.84±0.04	2.12±0.14 2.44±0.24	0.51 0.001	-0.98 -9.3	0.001
*p*-value^‡^	0.001	0.15			
**MDA (µM)**	4.64±0.81 2.99±0.22	4.12±0.57 5.63±0.67	0.60 0.001	0.74 -5.29	0.001
Before					
After					
*p*-value^‡^	0.054	0.14			
**CAT (KU)** Before After	5.24±0.51 6.98±0.37	6.63±0.53 6.86±0.41	0.06 0.83	-2.67 0.30	0.939
p-value^‡^	0.01	0.74			
**GPx (mU/ml)** Before After	242.87±2.61 250.75±1.11	243.87±2.41 246.65±1.33	0.79 0.02	-0.39 3.34	0.02
*p*-value^‡^	0.008	0.33			
**GSH (µM)**	4.42±0.56 23.94±2.63	5.54±0.71 15.20±1.79	0.22 0.008	-1.75 3.88	0.01
Before					
After					
*p*-value^‡^	0.001	0.001			
**TT (mM)** Before After	0.139±0.008 0.178±0.014	0.146±0.006 0.142±0.006	0.50 0.023	-0.99 3.34	0.01
*p*-value^‡^	0.018	0.48			
**FBS (mg/dl)** Before After	100.23±2.15 84.90±1.15	105.13±1.57 90.97±1.23	0.071 0.001	-2.60 -5.09	0.03
*p*-value^‡^	0.001	0.001			
**HOMA-IR** Before After	6.40±1.29 0.983±0.06	6.72±0.72 1.33±0.09	0.83 0.004	-4.53 -0.30	0.004
*p*-value^‡^	0.001	0.001			
**TG (mg/dl)** Before After	189.3±14.30 177.83±15.29	198.60±14.07 190.60±13.53	0.64 0.53	-0.88 -0.12	
					0.57
*p*-value^‡^	0.001	0.09			
**Chol (mg/dl)** Before After	198.66±7.10 174.36±6.89	199.43±4.51 187.96±4.70	0.93 0.11	-0.12 -2.30	0.016
*p*-value^‡^	0.001	0.002			
**HDL (mg/dl)** Before After	50.00±1.64 60.13±1.76	47.16±1.07 50.93±1.43	0.15 0.001	2.05 5.73	0.001
*p*-value^‡^	0.001	0.002			
**LDL (mg/dl)** Before After *p*-value^‡^	100.43±4.85 81.03±4.68 0.001	96.20±4.04 93.37±3.96 0.001	0.51 0.049	0.94 -2.84	0.001
**AST (U/l)** Before After	20.46±1.73 17.60±1.41	21.43±1.66 20.16±1.48	0.68 0.21	-0.57 -1.77	0.012
*p*-value‡	0.001	0.052			
**ALT (U/l)** Before After	22.73±1.77 18.70±1.39	22.20±1.64 24.00±1.62	0.83 0.016	0.31 -3.51	0.001
*p*-value^‡^	0.001	0.001			

Note: Results are presented as Mean±SEM. A significance level of 0.05 was considered (*p*-value<0.05).

† Independent t-test

‡ Paired t-test

§ Based on ANCOVA

Abbreviations: TAC: total antioxidant capacity; TOS: total oxidant status; OSI: oxidative stress index; MDA: malondialdehyde; CAT: catalase; GPx: glutathione peroxidase; GSH: reduced glutathione; TT: total thiol; FBS: fasting blood sugar ; HOMA-IR: homeostatic model assessment-insulin resistance; TG: triglyceride; Chol: cholesterol; HDL: high density lipoprotein; LDL: low density lipoprotein; AST: aspartate aminotransferase; ALT: alanine aminotransferase.

**Table 3:** Comparison of the effects of Vitex and placebo consumption on clinical characteristics of women with PCOS.

Variables	*Vitex* (n=30)	Placebo (n=30)	*p*-value^†^	Cohen’s d	*p*-value^§^
OV. Right (CC) Before After	14.58±0.75 12.06±0.53	13.75±0.73 12.30±0.54	0.43 0.76	1.12 -0.44	0.1
*p*-value^‡^	0.001	0.001			
OV. Left (CC) Before After	14.51±0.71 12.81±0.55	13.58±0.70 13.28±0.63	0.35 0.58	1.31 -0.79	0.023
*p*-value^‡^	0.001	0.47			
mFG Score Before After	10.70±0.70 7.83±0.58	9.20±0.58 11.26±0.69	0.11 0.001	2.33 -5.38	0.001
*p*-value^‡^	0.001	0.001			
Menstrual frequency Before After	0.200±0.06 0.955±0.02	0 .277±0.08 0.844±0.04	0.49 0.046	-1.08 3.51	0.027
*p*-value^‡^	0.001	0.001			

Results are presented as Mean±SEM. A significance level of 0.05 was considered (*p*-value<0.05).

† Independent t-test

‡ Paired t-test

§ Based on ANCOVA

OV: ovary volume; mF G: modified ferriman-gallwey.

Furthermore, values of TOS (*p*<0.001), OSI (*p*<0.001), MDA (*p*<0.001), FBS (*p*<0.001), HOMA-IR (*p*<0.05), ALT (0.05), mFG (0.001), and LDL (*p*<0.05) significantly decreased by the consumption of the *Vitex* compared to the placebo group. Values of TG, Chol, AST, right ovary volume, and left were decreased non-significantly in the *Vitex* group compared to the placebo group (*p*>0.05). In addition, CAT increased non-significantly in the *Vitex* group compared to the placebo group (*p*=0.83).

Based on ANCOVA, values of TAC (*p*<0.001), GPx (*p*<0.05), GSH (*p*<0.05), TT (*p*<0.05), HDL (*p<*0.001), and menstrual frequency (*p*<0.05) were significantly increased after the intervention compared to the placebo group. Furthermore, values of TOS (*p*<0.001), OSI (*p*<0.001), MDA (*p*<0.001), FBS (*p*<0.05), HOMA_IR (*p*<0.05), LDL (*p*<0.001), Chol (*p*<0.05), AST (*p*<0.001), ALT (*p*<0.001), ovary volume left (*p*<0.05). mFG score (*p*<0.001) were significantly decreased after supplementation compared to the placebo group. Consequently, the improvements observed in these variables may be attributed to the intervention, and after Covariance Analysis, AST, Chol, and left ovary volume showed a significant decrease. However, the ANCOVA for TG, CAT, and right ovary volume was non-significantly.

## DISCUSSION

This study assessed the effects of a 12-week intervention with *Vitex* on oxidative stress markers, insulin resistance, lipid profile, menstrual frequency, ovarian volume, and hirsutism in women with PCOS. While earlier studies have highlighted the benefits of *Vitex* on menstrual irregularities such as oligomenorrhea (Shayan *et al.*, 2016), this is the first clinical trial to systematically investigate its multi-dimensional effects across metabolic and clinical parameters in this population.

Our findings revealed that daily supplementation with 5.8 mg of *Vitex* extract significantly increased TAC, GPx, GSH, TT, and HDL-C, while decreasing TOS, OSI, MDA, FBS, HOMA-IR, total cholesterol, LDL-C, AST, and ALT levels compared to placebo. Additionally, it improved menstrual frequency, decreased left ovarian volume, and reduced hirsutism scores.

The observed improvements are likely mediated through *Vitex’s* rich phytochemical profile, particularly phenolic acids (e.g., vanillic acid) and flavonoids (e.g., quercetin), which possess antioxidant properties (Kavaz *et al.*, 2022).These compounds may enhance cellular antioxidant defense by upregulating enzymes such as GPx and GSH, mitigating oxidative damage that is known to impair insulin signaling and ovarian function (Zamora & Villena, 2014; Mohammadi, 2019).

*Vitex* may also exert endocrine effects by modulating dopamine D2 receptors, reducing prolactin secretion, which in turn supports menstrual regularity and ovulation (Feyzollahi *et al.*, 2021). Furthermore, it may regulate the hypothalamic-pituitary-gonadal axis via modulation of KISS-1 gene expression, a pathway implicated in GnRH pulpability and reproductive hormone balance (Puglia *et al.*, 2023). Its hypoglycemic and insulin-sensitizing effects may be partly attributed to inhibition of carbohydrate-hydrolyzing enzymes, α-amylase and α-glucosidase (Berrani *et al.*, 2021), reducing postprandial glucose excursions. These metabolic benefits may also lower circulating androgens, improving clinical manifestations such as hirsutism.

Our findings align with several animal studies reporting decreased MDA and improved antioxidant enzyme activity (SOD, CAT) following *Vitex* administration (Moreno *et al.*, 2015; Ahangarpour *et al.*, 2016). However, unlike these studies, we did not observe a significant increase in catalase activity, possibly due to the shorter intervention period or differences in assay sensitivity.

In terms of lipid profile, our results are consistent with Berrani *et al*. (2021), who demonstrated improved HDL-C and reduced TG and LDL-C levels in diabetic rats treated with *Vitex*. However, dosages varied widely across studies. While we used a low dose (5.8 mg), other trials in PMS and mastalgia have employed doses between 20-40 mg (Roemheld-Hamm, 2005) indicating that therapeutic efficacy may follow a non-linear dose-response pattern. Highlighted such variability, with low doses improving ovarian histology in animals while higher doses worsened it.

Despite promising results, several limitations must be acknowledged. First, the short duration (12 weeks) limits our ability to assess long-term effects on fertility or metabolic outcomes. Second, the single-center design and convenience sampling may reduce external validity, and although randomization minimized intergroup bias, the sample may not reflect the heterogeneity of PCOS phenotypes. Third, we did not stratify participants based on PCOS phenotype, BMI, or insulin resistance severity-factors that may modulate response to antioxidant therapy. Fourth, adherence to supplementation was self-reported, which could introduce recall bias. Fifth, the study lacked mechanistic biomarkers such as sex hormone-binding globulin (SHBG), LH/FSH ratio, or prolactin levels, which would further clarify hormonal effects.

The statistically significant findings also bear clinical importance. Improvements in antioxidant status and insulin resistance may help reduce long-term risks such as type 2 diabetes and cardiovascular disease. Reductions in hirsutism and irregular menstruation directly improve quality of life and reproductive health.

Compared to conventional interventions such as metformin and oral contraceptives, *Vitex* offers a complementary and potentially safer alternative, particularly for women seeking herbal therapies with fewer side effects. For instance, while metformin improves insulin sensitivity, it is often associated with gastrointestinal side effects (Kiani *et al.*, 2022). In contrast, *Vitex*, through its antioxidant and endocrine-modulating properties, not only improves insulin resistance but also addresses menstrual irregularities and hirsutism without reported major adverse events.

*Vitex* thus presents as a promising complementary therapy for women with PCOS, especially those seeking natural alternatives. Future trials should include larger, diverse populations, multiple dosing arms, longer durations, and mechanistic endpoints to elucidate *Vitex’s* full therapeutic potential-particularly in women planning pregnancy.

## CONCLUSIONS

This study’s outcomes demonstrate that *Vitex* supplementation effectively reduces oxidative stress and enhances antioxidant defense in women with PCOS. The significant improvements observed in insulin resistance, lipid profile, menstrual frequency, and clinical symptoms such as hirsutism suggest that *Vitex* may serve as a beneficial complementary therapy for managing PCOS-related complications. From a clinical standpoint, it is advisable for healthcare professionals to consider *Vitex* fruit extract-particularly in patients seeking natural alternatives-as part of an individualized treatment plan.

Future research should explore long-term effects, dose-response relationships, and its impact on hormonal biomarkers such as LH/FSH ratio and SHBG. Additionally, studies involving diverse PCOS phenotypes and stratified populations are needed to better understand the full therapeutic potential and safety profile of *Vitex* in reproductive and metabolic health.

## Abbreviations:

PCOS Polycystic ovary syndrome

IRInsulin resistance

VitexVitex agnus-castus

ANCOVA Analysis of covariance

BMI Body mass index

TTTotal thiol

TAC Total antioxidant capacity

FRAPFerric reducing ability of plasma

TOSTotal oxidant status

FBS Fasting blood sugar

TG Triglyceride

CholCholesterol

HDLHigh-density lipoprotein

LDL Low-density lipoprotein

AST Aspartate aminotransferase

ALT Alanine aminotransferase

ELISA Enzyme-linked immunosorbent assay

HOMA-IR Homeostasis model assessment

mFGModified Ferriman-Gallwey

## References

[r1] Ahangarpour A, Najimi SA, Farbood Y. (2016). Effects of Vitex agnus-castus fruit on sex hormones and
antioxidant indices in a D-galactose-induced aging female mouse
model. J Chinese Med Assoc.

[r2] Alois M, Estores IM. (2019). Hormonal Regulation In Pcos Using Acupuncture And Herbal
Supplements: A Case Report And Review Of The Literature. Integr Med (Encinitas).

[r3] Bannigida DM, Nayak BS, Vijayaraghavan R. (2020). Insulin resistance and oxidative marker in women with
PCOS. Arch Physiol Biochem.

[r4] Bargiota A, Diamanti-Kandarakis E. (2012). The effects of old, new and emerging medicines on metabolic
aberrations in PCOS. Ther Adv Endocrinol Metab.

[r5] Bednarska S, Siejka A. (2017). The pathogenesis and treatment of polycystic ovary syndrome:
What’s new?. Adv Clin Exp Med.

[r6] Berrani A, Marmouzi I, Bouyahya A, Kharbach M, El Hamdani M, El Jemli M, Lrhorfi A, Zouarhi M, Faouzi MEA, Bengueddour R. (2021). Phenolic Compound Analysis and Pharmacological Screening of Vitex
agnus-castus Functional Parts. Biomed Res Int.

[r7] Erel O. (2005). A new automated colorimetric method for measuring total oxidant
status. Clin Biochem.

[r8] Eyer P, Worek F, Kiderlen D, Sinko G, Stuglin A, Simeon-Rudolf V, Reiner E. (2003). Molar absorption coefficients for the reduced Ellman reagent:
Reassessment. Anal Biochem.

[r9] Feyzollahi Z, Mohseni Kouchesfehani H, Jalali H, Eslimi-Esfahani D, Sheik Hosseini A. (2021). Effect of Vitex agnus-castus ethanolic extract on hypothalamic
KISS-1 gene expression in a rat model of polycystic ovary
syndrome. Avicenna J Phytomed.

[r10] Kavaz A, Işık M, Dikici E, Yüksel M. (2022). Anticholinergic, Antioxidant, and Antibacterial Properties of
Vitex Agnus-Castus L. Seed Extract: Assessment of Its Phenolic Content by
LC/MS/MS. Chem Biodivers.

[r11] Kiani AK, Donato K, Dhuli K, Stuppia L, Bertelli M. (2022). Dietary supplements for polycystic ovary syndrome. J Prev Med Hyg.

[r12] Kiel IA, Lionett S, Parr EB, Jones H, Røset MAH, Salvesen Ø, Vanky E, Moholdt T. (2020). Improving reproductive function in women with polycystic ovary
syndrome with high-intensity interval training (IMPROV-IT): Study protocol
for a two-centre, three-armed randomised controlled trial. BMJ Open.

[r13] Li W, Liu C, Yang Q, Zhou Y, Liu M, Shan H. (2022). Oxidative stress and antioxidant imbalance in ovulation disorder
in patients with polycystic ovary syndrome. Front Nutr.

[r14] Masjedi M, Izadi Y, Montahaei T, Mohammadi R, Ali Helforoush M, Rohani Rad K. (2024). An illustrated review on herbal medicine used for the treatment
of female infertility. Eur J Obstet Gynecol Reprod Biol.

[r15] Minh HV, Tien HA, Sinh CT, Thang DC, Chen CH, Tay JC, Siddique S, Wang TD, Sogunuru GP, Chia YC, Kario K. (2021). Assessment of preferred methods to measure insulin resistance in
Asian patients with hypertension. J Clin Hypertens (Greenwich).

[r16] Mohammadi M. (2019). Oxidative Stress and Polycystic Ovary Syndrome: A Brief
Review. Int J Prev Med.

[r17] Moreira MV, Vale-Fernandes E, Albergaria IC, Alves MG, Monteiro MP. (2023). Follicular fluid composition and reproductive outcomes of women
with polycystic ovary syndrome undergoing in vitro fertilization: A
systematic review. Rev Endocr Metab Disord.

[r18] Moreno FN, Campos-Shimada LB, da Costa SC, Garcia RF, Cecchini AL, Natali MR, Vitoriano Ade S, Ishii-Iwamoto EL, Salgueiro-Pagadigorria CL. (2015). Vitex agnus-castus L. (Verbenaceae) Improves the Liver Lipid
Metabolism and Redox State of Ovariectomized Rats. Evid Based Complement Alternat Med.

[r19] Puglia LT, Lowry J, Tamagno G. (2023). Vitex agnus castus effects on hyperprolactinaemia. Front Endocrinol (Lausanne).

[r20] Roemheld-Hamm B. (2005). Chasteberry. Am Fam Physician.

[r21] Samimi F, Namiranian N, Sharifi-Rigi A, Siri M, Abazari O, Dastghaib S. (2024). Coenzyme Q10: A Key Antioxidant in the Management of
Diabetes-Induced Cardiovascular Complications-An Overview of Mechanisms and
Clinical Evidence. Int J Endocrinol.

[r22] Shayan A, Masoumi SZ, Shobeiri F, Tohidi S, Khalili A. (2016). Comparing the Effects of Agnugol and Metformin on Oligomenorrhea
in Patients with Polycystic Ovary Syndrome: A Randomized Clinical
Trial. J Clin Diagn Res.

[r23] Shokrpour M, Asemi Z. (2019). The Effects of Magnesium and Vitamin E Co-Supplementation on
Hormonal Status and Biomarkers of Inflammation and Oxidative Stress in Women
with Polycystic Ovary Syndrome. Biol Trace Elem Res.

[r24] Siddiqui S, Mateen S, Ahmad R, Moin S. (2022). A brief insight into the etiology, genetics, and immunology of
polycystic ovarian syndrome (PCOS). J Assist Reprod Genet.

[r25] Tahapary DL, Pratisthita LB, Fitri NA, Marcella C, Wafa S, Kurniawan F, Rizka A, Tarigan TJE, Harbuwono DS, Purnamasari D, Soewondo P. (2022). Challenges in the diagnosis of insulin resistance: Focusing on
the role of HOMA-IR and Tryglyceride/glucose index. Diabetes Metab Syndr.

[r26] Vale-Fernandes E, Moreira MV, Rodrigues B, Pereira SS, Leal C, Barreiro M, Tomé A, Monteiro MP. (2024). Anti-Müllerian hormone a surrogate of follicular fluid
oxidative stress in polycystic ovary syndrome?. Front Cell Dev Biol.

[r27] Vink JM, Sadrzadeh S, Lambalk CB, Boomsma DI. (2006). Heritability of polycystic ovary syndrome in a Dutch twin-family
study. J Clin Endocrinol Metab.

[r28] Wang Z, Qi F, Cui Y, Zhao L, Sun X, Tang W, Cai P. (2018). An update on Chinese herbal medicines as adjuvant treatment of
anticancer therapeutics. Biosci Trends.

[r29] Willis SK, Mathew HM, Wise LA, Hatch EE, Wesselink AK, Rothman KJ, Mahalingaiah S. (2020). Menstrual patterns and self-reported hirsutism as assessed via
the modified Ferriman-Gallwey scale: A cross-sectional study. Eur J Obstet Gynecol Reprod Biol.

[r30] Zamora M, Villena J. (2014). Targeting mitochondrial biogenesis to treat insulin
resistance. Curr Pharm Des.

[r31] Zeng X, Xie YJ, Liu YT, Long SL, Mo ZC. (2020). Polycystic ovarian syndrome: Correlation between
hyperandrogenism, insulin resistance and obesity. Clin Chim Acta.

